# A method for determining cell number in the undisturbed epithelium of the mouse lens

**Published:** 2010-11-04

**Authors:** Steven Bassnett, Yanrong Shi

**Affiliations:** Department of Ophthalmology and Visual Sciences, Washington University School of Medicine, St. Louis, MO

## Abstract

The anterior face of the mouse lens is covered by a layer of epithelial cells. The epithelial cells serve a barrier function at the lens surface and as a progenitor population from which lens fiber cells, the predominant cell type of the lens, are derived. Decreased epithelial cell density is commonly observed during aging and cataract formation in humans and animal models and may contribute directly to tissue opacification. However, the loss of cells from the epithelium is often not easy to quantify, in part because the cells are arrayed across a near-spherical surface and, as a consequence, are difficult to image and count. Here, we describe a technique for determining epithelial cell number in the undisturbed lens of the mouse, a popular cataract model. The method utilizes orthographic projections of confocal images collected from the anterior and equatorial regions of the lens. The overlapping projections are brought into register using the unique distribution of proliferating cells as fiduciary points. Cell counts are performed using a computer-assisted method. This approach offers several advantages over flat-mount methods employed previously.

## Introduction

The lens epithelium serves a barrier function and also provides progenitor cells from which lens fibers, the predominant cell type of the lens, are derived. In the lenses of many species, marked decreases in epithelial cell density are observed during normal aging [1] or accompanying cataract formation induced by X-irradiation [2], gene mutation [3], diabetes [4], UV exposure [5], or other cataractogenic agents. The mouse is a popular model for studying mechanisms underlying cataract formation. Here, we describe a method for accurately determining the number of epithelial cells in the undisturbed mouse lens.

The adult mouse lens approximates an oblate spheroid with an equatorial diameter of 2–3 mm. Accurately counting epithelial cells on the large, curved anterior surface of the lens is technically challenging. Previous studies on rodent lenses [6-8] circumvented this problem by performing cell counts on flat-mounts prepared from the epithelium. In this technique, the epithelium is carefully dissected from the lens and pinned to the base of a Petri dish. After appropriate staining to visualize individual cells, representative areas are counted to establish cell density. The surface area of the anterior hemisphere is calculated from a knowledge of the lens diameter (generally with the simplifying assumption that the mouse lens is spherical) and this value is then combined with the cell density measurements to calculate the total number of cells in the epithelium.

Although technically straightforward, the flat-mount approach has several shortcomings. First, manual dissection of the epithelium excludes or damages cells located near the periphery of the epithelium. Second, a curved surface cannot be flattened without distortion. Thus, during preparation of epithelial flat-mounts the epithelium must either be cut or stretched (or both) to pin it flat in the dish. Third, cell density is not uniform across the epithelium. Any estimate based on a regional cell count will, therefore, be erroneous.

In our approach, cells are visualized and counted in situ in the intact lens epithelium. This strategy has the further advantage that the spatial relationships between neighboring epithelial cells and between epithelial cells and the underlying fiber cells are retained.

## Methods

### Nuclear labeling

Nuclei were counted as surrogates for the epithelial cells. Nuclei were visualized following labeling with 5-ethynyl-2´-deoxyuridine (EdU; Invitrogen, Carlsbad, CA) and/or Draq5 (Biostatus Ltd, Leicester, UK). EdU is a thymidine analog and incorporated into newly-synthesized DNA during S-phase of the cell cycle. For EdU labeling, 8-week-old C57/Bl6 mice were given an intra-peritoneal injection of EdU (10 µg/g bodyweight), as described [9]. One hour after injection, mice were killed by CO_2_ inhalation and lenses were dissected from the eye, fixed for 1 h in 3.7% formaldehyde/PBS, and permeabilized in 0.5% Triton X-100. EdU-positive cell nuclei were visualized using Click-iT™ (Invitrogen) chemistry, which features a copper-catalyzed, covalent reaction between an alkyne group in the EdU and an azide group in an Alexa 488 fluorochrome. Lenses were incubated in 1× Click-iT reaction cocktail for 30 min. Lenses were also counterstained with the far red fluorescence DNA dye, Draq5. Lenses were stained for 30 min at room temperature in a 1:1,000 dilution of Draq5 in PBS. These procedures were approved by the Washington University Animal Studies Committee.

### Microscopy and analysis

**Figure 1:**
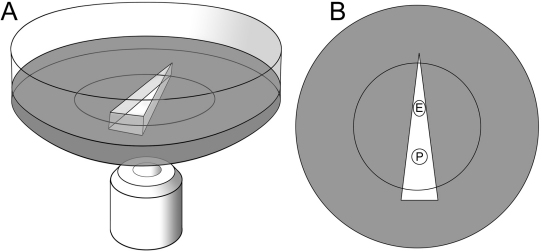
Glass-bottomed chamber for positioning and viewing mouse lenses. Oblique view (**A**) and a plan view (**B**). The base of the chamber contains a layer of solidified agar from which a wedge-shaped piece has been removed. To image the anterior pole (P), the lens, resting on its epithelium, is positioned in the base of the wedge. To visualize the equatorial region (E), the lens is turned on its side and positioned such that its anterior and posterior faces are supported by the gently tapering walls of the wedge and the equatorial cells are exposed.

Lenses were viewed in glass-bottomed microwell dishes (Cat# P35G-1.5–10-C; Mattek, Ashland, MA). The chambers were prefilled with a shallow layer of molten agarose (4% in tissue culture medium). Once the agarose had cooled and set, a wedge-shaped piece was removed using a scalpel, to provide a viewing chamber (Figure 1). The chamber was then filled with pre-warmed tissue culture medium (DMEM/F12; Invitrogen) and a mouse lens maneuvered into position. The anterior and equatorial aspects of the lens were imaged independently. Lenses from 8-week-old mice are ≈2.5 mm in equatorial diameter. The field of view of the 10× objective used for most imaging studies was 1.15 mm×1.15 mm. To image the anterior pole, the lens was positioned in the wide end of the wedge and allowed to rest, face down, on its epithelium. For equatorial imaging, the lens was turned on its side and positioned such that the anterior and posterior lens faces were gently supported by the tapering walls of the wedge (Figure 1B).

The lenses were viewed using a confocal microscope (LSM510 META; Zeiss, Thornwood, NY) in the inverted configuration. A plan-apochromat 10× objective lens (0.45 NA) or a 40× C-apochromat objective (1.2 NA) were used to collect the image stacks. EdU-labeled nuclei were detected using the 488 nm line of an argon laser and a 505–550 nm band-pass emission filter. Draq5-stained nuclei were imaged using the 633 nm laser line and a 650 nm long-pass emission filter.

## Results and discussion

**Figure 2:**
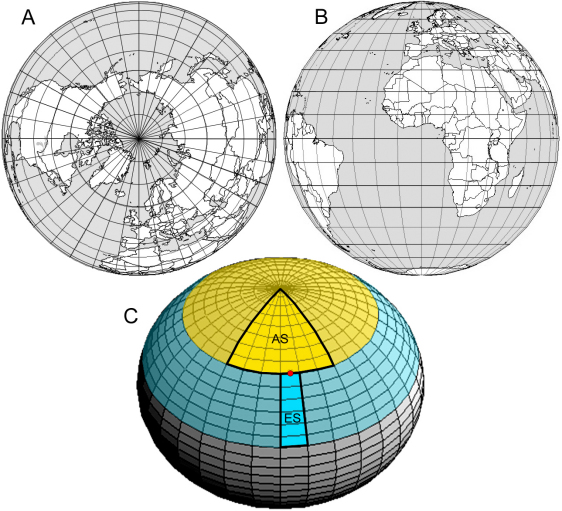
Orthographic projections. **A**: Orthographic azimuthal projection of the globe, polar aspect. **B**: Orthographic azimuthal projection of the globe, equatorial aspect. **C**: Orthographic projection of a lens, showing an oblique view of the anterior surface. The epithelium is arbitrarily divided into two regions: a spherical cap (yellow) and an equatorial band (blue). The number of cells in the spherical cap is calculated from cell counts made on a 60°-wide anterior sector (AS). The number of cells in the equatorial band is determined by cell counts made on a 10°-wide equatorial sector (ES). The border between AS and ES is marked by the position of a fiduciary, EdU-positive nucleus (red dot). See text for details.

Image stacks of the lens anterior surface and equatorial region were collapsed to two-dimensional, maximum intensity projections. Such projections are equivalent to orthographic azimuthal projections used by cartographers to depict the earth as seen from a great distance. Orthographic projections have the following features: in the polar aspect (Figure 2A), lines of longitude are straight and lines of latitude form a series of concentric circles. In the equatorial aspect (Figure 2B), lines of latitude are parallel and lines of longitude appear increasingly curved as one moves away from the center of the projection.

For our analysis, we divided the lens epithelium into two regions: a spherical cap and an epithelial band (colored yellow and blue, respectively, in Figure 2C). The number of cells in the two regions was determined independently, from orthographic projections of the anterior and equatorial regions. The two projections, which were oriented orthogonally to each other, were brought into registration by identifying a fiduciary point, an EdU-positive nucleus, clearly visible in both projections. One such nucleus is shown in diagrammatic form in Figure 2C. The lens is radially symmetric about the optical axis. It is not necessary, therefore, to count every cell in either the spherical cap or the equatorial band. With regard to the latter, we counted cells in a 10°-wide equatorial sector (ES, in Figure 2C) in the center of the projection, located between the equatorial margin of the epithelium and the fiduciary cell. Confining the analysis to a relatively small region of the equatorial surface greatly simplified the measurement geometry: under these circumstances, ES closely approximates an isosceles trapezoid.

**Figure 3:**
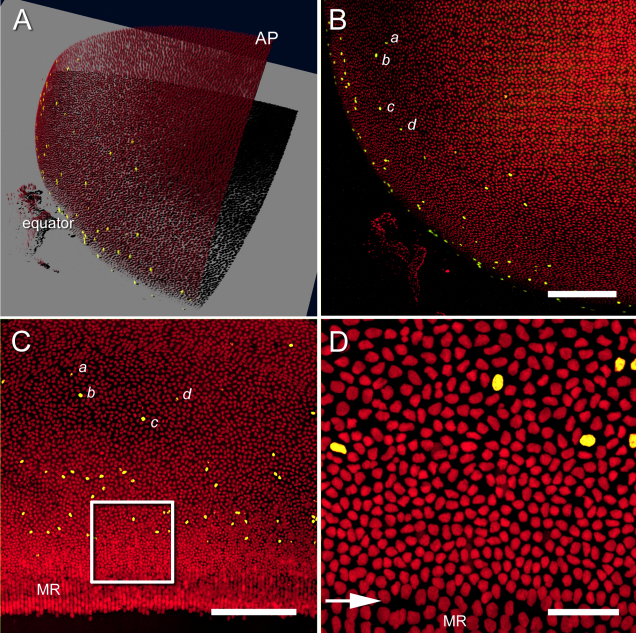
Orthographic projections of anterior or equatorial regions of the mouse lens. **A**: Three-dimensional rendering of an anterior lens quadrant, showing EdU-positive nuclei (yellow) and total draq5-stained nuclei (red). An animated version of this panel can be viewed at animation1. EdU-positive cells are relatively numerous near the lens equator but rare or absent in the anterior polar (AP) region. **B**: Two-dimensional orthographic projection of the data shown in **A**. The EdU-positive cells form recognizable “constellations.” One such constellation is formed by four nuclei: a, b, c, d. **C**: Two-dimensional orthographic projection of the lens equator. The constellation a, b, c, d, is also discernible in this orientation. **D**: High magnification view of the boxed region shown in **C**. A change in the size and orientation of the fiber cell nuclei (arrow) indicates that fiber cell differentiation has commenced and that cells have entered the meridional rows (MR). Scale bars in **B** and **C** are 250 µm, and in **D** is 50 µm.

Stacks of optical sections of EdU- and Draq5-stained lenses were collected from the anterior (Figure 3A) or equatorial region and then converted into anterior (Figure 3B) or equatorial (Figure 3C) orthographic projections using Metamorph Image Analysis software (Verson 7.7 ; Molecular Devices, Downingtown, PA). An animated version of Figure 3A can be viewed by clicking animation1. As expected, EdU-positive nuclei were most numerous near the lens equator (Figure 3A), in the so-called “germinative zone,” a region of high mitotic activity [10]. Clusters of Edu-positive nuclei in this region often formed recognizable patterns, or “constellations,” that were apparent in projections generated from both polar and equatorial image stacks. Nuclei within these constellations were selected as fiduciary points, delineating the border between the AS and ES (see Figure 2). In the vicinity of the lens equator, cell nuclei became more oval in shape, less densely packed and organized in regularly-spaced columns (Figure 3D). These radially-oriented columns have been termed meridional rows. Previous studies have sometimes included the meridional rows in epithelial cell counts (see, for example [6,7], ). However, based on our own observations [11], we consider cells of the meridional rows to be young fiber cells and they are excluded from our cell counts.

**Figure 4:**
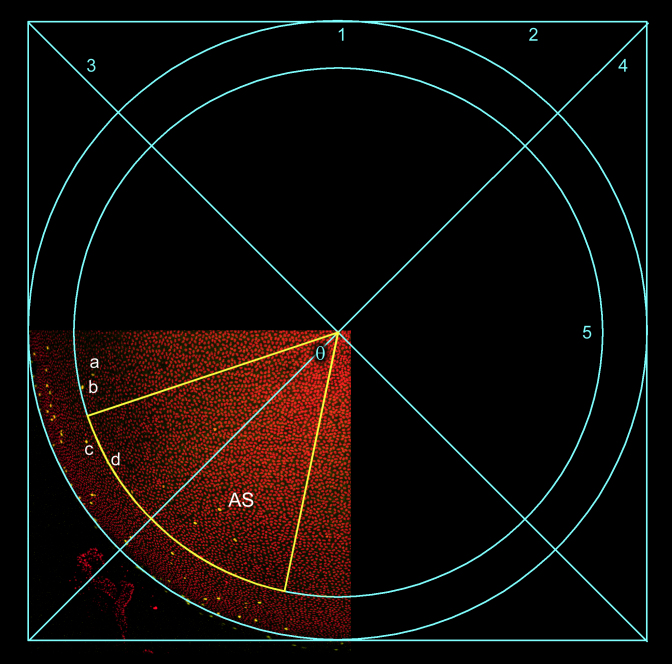
Defining the anterior sector (AS). An orthographic projection of an anterior lens quadrant is generated. A circle (1) of diameter D is fitted by eye to the arc of the quadrant. A square (2) with sides equal to D is fitted around the circle. The intercept of diagonals (3 and 4) defines the center of the circle. The fiduciary nucleus, *d*, in the constellation *a*, *b*, *c*, *d* is identified in the projection. The distance between *d* and the center of the circle is measured and used as the radius of a second, smaller circle (5), concentric with the first. An anterior sector (yellow) is drawn in the smaller circle, with a central angle (θ) of 60°.

**Figure 5:**
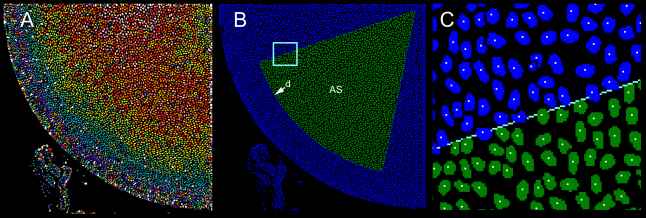
Computer-assisted identification and quantification of nuclei in a 60° sector (AS) of the anterior lens epithelium. **A**: Nuclei are identified using the count nuclei application. Colors indicate nuclear size (large nuclei are shown in warmer colors). **B**: Following image segmentation, nuclei lying within sector AS are counted (green). The arc of the sector is located at d, the fiduciary nucleus (see Figure 3 and Figure 4). **C**: A higher magnification view of the boxed area in **B**. The position of the centroid (center of mass) of each nucleus is calculated (indicated by a white dot). Only nuclei with centroids located within AS are included in the analysis.

A sector with a central angle of 60° was defined on the anterior orthographic projection (Figure 4). Nuclei were identified automatically using the *count nuclei* application in Metamorph (Figure 5A). This application uses the expected size and brightness of nuclei to discriminate them against a potentially fluctuating background. The image was segmented and the number of nuclei within the measurement sector, AS, determined (Figure 5B). For each nucleus, the position of the centroid (center of mass) was calculated (Figure 5C). Only nuclei with a center of mass located within the measurement sector were counted. Typically, the number of nuclei in the anterior sector of an 8-week-old mouse lens was about 2,500. Multiplying this value by six (360/60) gives a value of 15,000 epithelial cells in a typical spherical cap from an 8-week-old lens.

**Figure 6:**
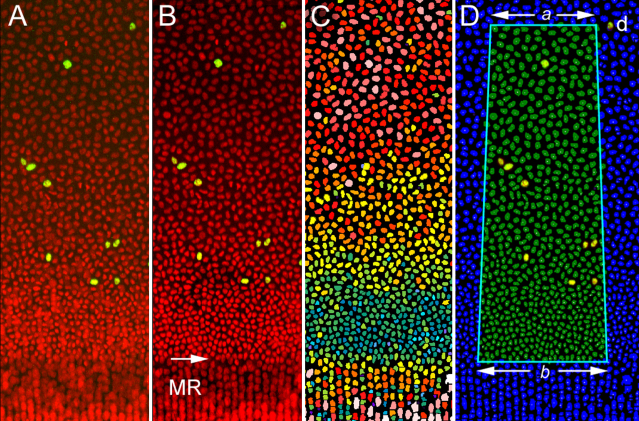
Quantification of epithelial nuclei in orthographic projections of the equatorial lens region. **A**: Projection of double-labeled lens tissue showing Edu-positive nuclei (yellow) and Draq5-stained nuclei (red). **B**: Three-dimensional editing of the original image stack removes the underlying fiber cell nuclei and clarifies epithelial nuclear fluorescence, facilitating automated counting. An arrow indicates the border between the epithelial cell nuclei and those of young fiber cells in the meridional rows (MR). **C**: Epithelial nuclei are identified automatically and color-coded according to size. Note the densely packed nuclei (blue green color) near the border of the epithelium. **D**: An isosceles trapezoid is defined in the center of the projection. The width of the trapezoid corresponds to 10° of longitude (See Figure 2). Sides *a* and *b* are oriented parallel to the lens equator. Side *a* is positioned level with the fiduciary cell, *d* (see Figure 4) and side *b* is located at the border between the epithelium and the meridional row cells. Nuclei (green) with centroids lying within the trapezoid are included in the count.

To determine the number of epithelial cells in the equatorial band, orthographic projections were generated of the lens equator. Even using confocal optics it was not possible to completely exclude fluorescence emanating from fiber cell nuclei located immediately below the equatorial epithelium (Figure 6A). The fiber fluorescence degraded the image quality in this region and made automated counting of epithelial nuclei problematic. To correct this problem, fiber cell nuclei were manually deleted in optical sections located beneath the plane of the equatorial epithelium. This resulted in a much improved signal-to-noise ratio in the epithelial layer (compare the equatorial region in Figure 6A,B). An isosceles trapezoid was defined on orthographic projections of the lens equator. The trapezoid spanned 10° of longitude in the center of the projection. The base of the trapezoid was located at the epithelial margin and the top of the trapezoid was aligned with the fiduciary nucleus.

Epithelial nuclei were defined with the *count nuclei* application (Figure 6C), the centroid of each nucleus was computed and those nuclei with centroids located within the measurement trapezoid were counted. In lenses from 8-week-old mice the number of cells in a typical equatorial sector was about 700 (depending on the location of the fiduciary cell). The equatorial band thus contained 25,200 cells (700×360/10). The total number of epithelial cells in the lens epithelium was calculated by summing the values for the anterior spherical cap and the equatorial band. For lenses from three 8-week-old animals the value was 44,429±2,850 (mean±SD, n=3). The technique was reasonably reproducible. In four cases, two independent measurements were made on the same lens. Thus, different quadrants of the lens were imaged, different projections were generated and different fiduciary cells were selected. The difference in calculated cell number between two replicate measurements was 8.2±4.6% (mean±SD, n=4). The variability in the measurements may in part reflect radial non-uniformities in cell density. The method described here samples only a fraction of the total epithelial area. If the sample is not truly representative of the whole then errors will be magnified, particularly in the equatorial measurements where only 1/36 of the surface is analyzed.

When examining the impact of various agents on the lens epithelium, previous studies have tended to restrict their analysis to effects on cell density, usually measured in the readily accessible central epithelium. However, some studies have examined the epithelial cell population as a whole. For example, two previous studies reported values of 40,661 [7] and 44,474 [6] for total cell number in the lens epithelium of 8-week-old mice (the same age examined in the current study). The latter value, from the Rafferty laboratory [6], explicitly includes 8,913 cells of the meridional rows. The value of 40,661, obtained by von Sallmann [7], probably also includes meridional row cells, as judged by earlier methodological references from that laboratory [12]. As mentioned earlier, we consider the meridional rows to be composed of young fiber cells rather than bona fide epithelial cells. Consequently, meridional row cells were not included in our cell counts. Subtracting the meridional row cell numbers from the earlier estimates gives values of 31,748 and 35,561,respectively; both values being considerably lower than the value of 44,429 obtained from the current work. The most likely explanation for this discrepancy is the use of flat-mount preparations in the earlier studies. The flat-mount technique requires the epithelium to be dissected from the lens. Although the central epithelium is usually well preserved during this procedure, the epithelial margin is inevitably damaged. As shown in Figure 6C, the margin of the epithelium contains the highest density of cells and damage to this region will likely result in significantly undercounting the total epithelial cell number.

### Summary

The mouse lens epithelium consists of 40,000 to 50,000 cells arrayed across the anterior surface of the lens. The curvature and relatively large surface area of the lens epithelium poses several challenges when seeking to accurately count the cells. Here, we used orthographic projections of the equatorial and anterior regions for cell counting. The epithelial cell field is devoid of features or physical landmarks that might be used to bring the equatorial and anterior projections into registration. To overcome this difficulty, we used unique and readily identified patterns of proliferating cells (constellations), visualized following EdU labeling, as fiduciary points to align the projections. Estimates of epithelial population number derived using our method are significantly higher than published values, perhaps as a result of more accurate counting of the densely-packed cells near the lens equator. The methodology should be applicable to any analysis of epithelial cell number or mitotic activity and could readily be extended to the lenses of other species, including humans.

## Supplementary Material

Supporting Movie
